# Prevention and Control of Diseases at the Interface of Livestock, Wildlife and Humans

**DOI:** 10.3390/vetsci6010011

**Published:** 2019-01-22

**Authors:** Richard Kock, Cord Heuer

**Affiliations:** 1 The Royal Veterinary College, University of London, Hawkshead Lane, North Mymms, Hatfield, Hertfordshire AL9 7TA, UK; 2 Institute of Vet, Animal and Biomedical Sciences, Massey University, Private Bag 11 222, Palmerston North 4442, New Zealand; C.Heuer@massey.ac.nz

Relatively few scientists are investigating health at the wildlife–livestock interface. Based on the membership in the Wildlife Disease Association with sections in North and South America, Europe, Africa, Australasia, and Asia, there are roughly 1500 dedicated wildlife health and disease specialists globally. However, only a few veterinarians, epidemiologists and public health specialists focus on the interface between wildlife and domestic animal species, a drop in the ocean of health professionals. More professionals are involved in the public health aspects of interactions between humans and domestic animals. As we argue below, the wildlife–domestic animal interface becomes increasingly important for environmental conservation, biosecurity, livestock production and health, and eventually human health. The theme of this Special Issue is therefore highly topical and timely.

As scientists, we need to be concerned about the relationships between human and natural landscapes. Some 40% of the terrestrial land area of the Earth is dedicated to animal production. This contributes about 75% of the global animal biomass, primarily livestock and poultry and, to a lesser extent, marine animals. Wildlife populations and species diversity are under immense threat and decrease rapidly under the intense pressure of human expansion in almost every ecologic entity of the globe. At the opposite end, the decline in wildlife species is associated with a rapid increase and dispersion of pathogenic microbial communities. In fact, it appears certain microbes are capitalising on this change, by thriving and adapting to new hosts, apparently without being sufficiently constrained or buffered by biological competition and evolving biodiversity. In the past, diverse and resilient natural ecosystems have limited the emergence of pathogens due to competing non-pathogenic microbes. Such a process favours ecologic stability and the maintenance of diverse ecosystems. It reduces the risk of new emerging pathogens that might otherwise lead to epi- or pandemics. Clear evidence exists already that disregarding the decline of biodiversity and natural ecosystems can give rise to negative impacts on public health and human well-being. Climate change accelerates the emergence of environmentally-derived infectious pathogens and even the development of non-communicable diseases and their associated socioeconomic burden. A more favourable public policy and a shift in funding allocation are desired changes that would motivate a larger number of professionals and academics to start working in this area. More scientific work is required to challenge current myths and presumptions that these imminent ecological changes are rather trivial. Direct infection of livestock and humans from wildlife was hitherto regarded to be a rare event. However, the experience of highly pathogenic avian influenza (HPAI), leptospirosis, the continued dispersion of Hanta and West Nile virus, and viral haemorrhagic fevers such as Lassa and Ebola and many other examples have taught us otherwise. It is important to understand the drivers of these microbial switches, leading to so-called pathogen jumps and how they are generated through human activities and medium- and long-term ecological changes. Whereas wildlife is commonly seen as a threat to human and animal health, evidence suggests quite the opposite: The relatively few surviving populations of wild animal species are struggling to cope with emerging diseases that spill over from domestic animals and people to wildlife such as toxoplasmosis and cryptosporidiosis.

Papers in this series cover a large diversity of topics. The dog sentinel study demonstrates the One Health dimension of disease [1]. It is an example of the value of animals as sentinel indicators of infection, beneficial to humans and across animal sectors. The essays about brucellosis show how these cryptic bacteria have survived and adapted to new landscapes and animal communities in a number of concerning ways [2,3]. The methodology for investigating the wildlife–animal interface is lacking scientific rigor. Mathematical modelling may be a way to fill this gap [4]. The paper addressing this aspect describes simple and complex approaches to measure and evaluate multi-species interactions. Such approaches, however, may not easily be understood by managers, stakeholders and political decision-makers engaged in the governance of developing and propagating solutions [5].
